# Mechanisms of Ferroptosis and Emerging Links to the Pathology of Neurodegenerative Diseases

**DOI:** 10.3389/fnagi.2022.904152

**Published:** 2022-06-28

**Authors:** Yiyan Sun, Xiaohuan Xia, Diksha Basnet, Jialin C. Zheng, Jian Huang, Jianhui Liu

**Affiliations:** ^1^Department of Anesthesiology, Tongji Hospital Affiliated to Tongji University School of Medicine, Shanghai, China; ^2^Center for Translational Neurodegeneration and Regenerative Therapy, Tongji Hospital Affiliated to Tongji University School of Medicine, Shanghai, China; ^3^Shanghai Frontiers Science Center of Nanocatalytic Medicine, Shanghai, China; ^4^Translational Research Institute of Brain and Brain-Like Intelligence, Shanghai Fourth People’s Hospital Affiliated to Tongji University School of Medicine, Shanghai, China; ^5^Collaborative Innovation Center for Brain Science, Tongji University, Shanghai, China; ^6^Key Laboratory of Systems Biomedicine (Ministry of Education) and Collaborative Innovation Center of Systems Biomedicine, Shanghai Center for Systems Biomedicine, Shanghai Jiao Tong University, Shanghai, China

**Keywords:** ferroptosis, neurodegenerative diseases, iron metabolism, oxidative stress, redox regulation

## Abstract

Neurodegenerative diseases are a diverse class of diseases attributed to chronic progressive neuronal degeneration and synaptic loss in the brain and/or spinal cord, including Alzheimer’s disease, Parkinson’s disease, Huntington’s disease, amyotrophic lateral sclerosis and multiple sclerosis. The pathogenesis of neurodegenerative diseases is complex and diverse, often involving mitochondrial dysfunction, neuroinflammation, and epigenetic changes. However, the pathogenesis of neurodegenerative diseases has not been fully elucidated. Recently, accumulating evidence revealed that ferroptosis, a newly discovered iron-dependent and lipid peroxidation-driven type of programmed cell death, provides another explanation for the occurrence and progression of neurodegenerative diseases. Here, we provide an overview of the process and regulation mechanisms of ferroptosis, and summarize current research progresses that support the contribution of ferroptosis to the pathogenesis of neurodegenerative diseases. A comprehensive understanding of the emerging roles of ferroptosis in neurodegenerative diseases will shed light on the development of novel therapeutic technologies and strategies for slowing down the progression of these diseases.

## Introduction

Dolma et al. (2003) discovered a new mechanism of cell death using a small molecule called erastin. In 2012, Scott Dixon discovered that erastin caused a non-apoptotic form of cell death with unique morphological, biochemical, and genetic properties, which was later termed ferroptosis (Dixon et al., 2012). As our knowledge of cell death continues to be updated, the concepts and mechanisms of ferroptosis are further elucidated. In 2018, the Nomenclature Committee on Cell Death defined ferroptosis as a Regulated Cell Death (RCD) caused by oxidative distress in the intracellular micro-environment, which can be inhibited by lipophilic antioxidants and iron chelators (Galluzzi et al., 2018). Recently, numerous studies have shown that ferroptosis is closely related to various diseases, including tumors (Mou et al., 2019), neurological disorders (Ren et al., 2020), metabolic diseases (Le et al., 2021), and cardiovascular diseases (Yu et al., 2021).

Neurodegenerative diseases (NDDs) are a group of diseases characterized by the progressive loss of a specific population of neurons, resulting in progressive cognitive decline, movement impairment, and other comorbidities, including Alzheimer’s disease (AD), Parkinson’s disease (PD), Huntington’s disease (HD), amyotrophic lateral sclerosis (ALS), multiple sclerosis (MS), and so on (Wells et al., 2019). Ferroptosis has been proven to be closely correlated with the occurrence and development of most NDDs. Here, we introduce the molecular mechanism of ferroptosis, the contribution of ferroptosis to the pathogenesis of NDDs, and the potential value of targeting ferroptosis in the treatment of NDDs.

## The Process and Regulation of Ferroptosis

### The Process of Ferroptosis

**FIGURE 1:**
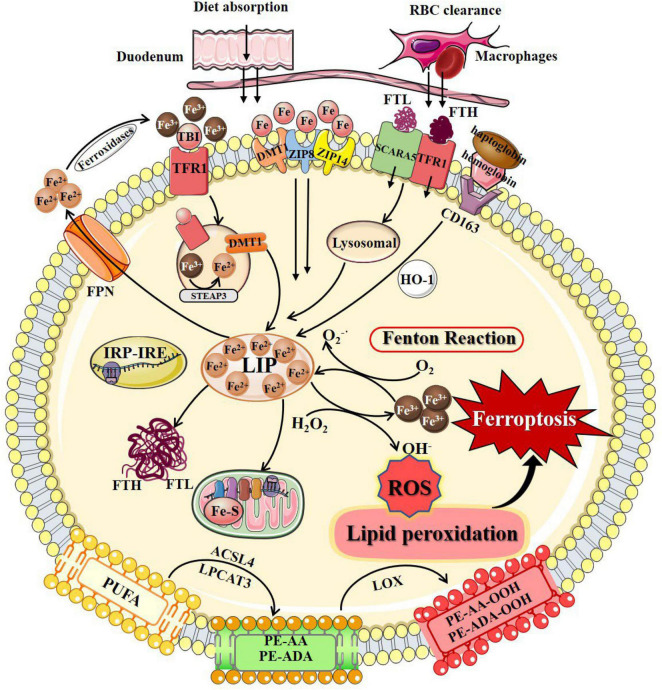
The process of ferroptosis. The occurrence of ferroptosis depends on the intracellular iron deposition caused by the disorder of iron metabolism. The body iron intake takes place by tissue macrophage-dependent aging red blood cells engulfing and duodenal enterocytes-mediated diet absorption into the bloodstream. Cellular iron absorption involves the following pathways: transferrin-bound iron pathway, non-transferrin bound iron pathway, SCARA5/TfR1-dependent endocytosis, and hemoglobin-dependent pathway. Intracellular Fe^2+^ formed LIP, stored in FTL and FTH in the cytosol, or as Fe–S in the mitochondrial respiratory chain. IRP/IRE regulates iron homeostasis by regulating the transcription of iron metabolism genes. When cellular iron metabolism is disordered, accumulated intracellular Fe^2+^ undergo Fenton’s reaction with H_2_O_2_ to generate OH^⋅^. OH^⋅^ and other ROS attack the PUFAs on the membrane surface to cause membrane peroxidation and release oxidative toxic substances such as 4-HNE and MDA. Consequently, membrane structure, proteins, and genetic materials are damaged, which affects organelles’ functions and cell homeostasis, causing ferroptosis at last. 4-HNE, 4-hydroxynonenal; ACSL4, long-chain-fatty-acid-CoA ligase 4; DMT1, divalent metal transporters 1; FPN, ferroportin; Fe–S, iron-sulfur clusters; FTH, ferritin heavy chain; FTL, ferritin light chain; HO-1, heme oxygenase 1; IRP/IRE, iron-regulatory protein/iron-responsive element; LIP, labile iron pool; LPCAT3, lysophosphatidylcholine acyltransferase 3; LOX, lipoxygenase; MDA, malondialdehyde; PUFAs, polyunsaturated fatty acids; PE-AA, phosphatidylethanolamines-arachidonoyl; PE-AA-OOH, hydroperoxides of phosphatidylethanolamines-arachidonoyl; PE-ADA, phosphatidylethanolamines-adrenoyl; PE-ADA-OOH, hydroperoxides of phosphatidylethanolamines- adrenoyl; ROS, reactive oxygen species; SCARA5, scavenger receptor class A member 5; TfR1, transferrin receptor 1; ZIP8, Zrt/Irt-related protein 8; ZIP14, Zrt/Irt-related protein 14.

As an independent form of cell death, ferroptosis can be clearly distinguished from other forms of cell death by the accumulation of lethal reactive oxygen species (ROS) and lipid peroxidation products caused by iron-dependent reactions (Table 1; Dixon et al., 2012; Wu et al., 2012; Liu and Levine, 2015; Chen et al., 2016; Xie et al., 2016; Galluzzi et al., 2017; D’Arcy, 2019; Xu et al., 2019; Demarco et al., 2020; Mizushima and Levine, 2020; Nah et al., 2020; Khan et al., 2021; Li et al., 2021; Nadeem et al., 2021; Yang et al., 2021; Braicu et al., 2022; Lei et al., 2022). Ultrastructural changes in mitochondria are the most prominent morphological features of ferroptotic cells. Overall, the process of ferroptosis consists of multiple key steps including iron metabolism dysregulation and lipid peroxidation (Figure 1).

**TABLE 1:** The main features of ferroptosis, apoptosis, autophagy, necroptosis, and pyroptosis.

		Ferroptosis	Apoptosis	Autophagy	Necroptosis	Pyroptosis
Morphological features	Cell morphology	Smaller and rounder; cell rounding up	Shrinkage; intercellular connections disappear	Minor changes	Swelling	Swelling; formed bubble-like protrusions
	Cell membrane	No rupture or blistering	Plasma membrane blebbing; membrane structure remains intact	Blebbing sometimes observed	Rupture of plasma membrane	Formation of plasma membrane pores; plasma membrane rupture
	Cytoplasm	Small mitochondria with condensed mitochondrial membrane densities	Retraction of pseudopods; dense cellular contents	Vacuolization of the cytoplasm; accumulation of double-membraned autophagic vacuoles	Cytoplasmic swelling, swelling of cytoplasmic organelles	Osmotic swelling; cell contents leakage
	Nucleus	Normal nuclear size; lack of chromatin condensation	Genetic materials fragmentation; marked chromatin condensation; nuclear fragmentation and condensation	Lack of chromatin condensation	Mild-moderate chromatin condensation (Nuclear pyknosis)	Chromatin random breakage degradation
	Special features	Mitochondrial atrophy or fragmentation; mitochondrial membrane density condensed; mitochondrial cristae decreased	Apoptotic bodies (or ApoBDs)	Numerous autophagosomes and autolysosomes	Necroptotic bodies	Pyroptotic bodies
Biological features		Iron and ROS accumulation; lipid peroxides increased; system Xc^–^ and GPX4 inhibition; GSH depletion; Δψm dissipation	Caspase activation; DNA fragmentation; Δψm dissipation; intracellular calcium increased	LC3-I to LC3-II conversion; ATG expression increased; increased lysosomal activity	Activation of RIP1, RIP3, and MLKL; PARP1 hyperactivation; drop in ATP levels	Dependent on caspase-1; GSDMD family activation
Immunological features		Release of DAMPs (e.g., inflammatory factor, arachidonic acid mediators, HMGB1)	Release Ecto-CRT, Histone, HMGB1, and ATP under certain conditions	Regulation of immune cell differentiation and function	Release of DAMPs (e.g., DNA, IL-6 and HMGB1)	Release of proinflammatory cytokine
Inflammation		Pro-inflammatory	Anti-inflammatory	Mostly anti-inflammatory	Mostly pro-inflammatory	Pro-inflammatory
Major regulatory components		P53, HO-1, iron, systemXC-/GSH/GPX4 pathway, GCH1/DHFR/BH4 pathway, FSP1/CoQ10 pathway, DHODH/CoQ pathway, p62/Keap1/Nrf2 pathway	P53, Bax and other Bcl-2 family proteins, Caspase family, endoplasmic reticulum pathway, death receptor	ATG family proteins (e.g., Atg5 and Atg7), Beclin 1, PI3K-AKT-mTOR pathway, MAPK-ERK1/2-mTOR pathway	Toll-like receptor family, RIP1, RIP3, MLKL, TNF-α, TRAIL, FasL, ROS	Caspase-1, Caspase-4/5/11, GSDMD, IL-1β, IL-18, NLRP3-mediated signaling pathway
Main inducer and inhibitor	Inducer	Erastin, sulfasalazine, sorafenib, altretamine, RSL3, ML162, ML210, SAS, lanperisone, DPI7, DPI10, FIN56, CIL56, artemisinin, FINO_2_	Ca^2+^/Mg^2+^, TGF-β glucocorticoid, FASL	Rapamycin, lithium, sodium, brefeldin A, thapsigargin, tunicamycin valproate, carbamazepine, xestospongin B/C, C2-ceramide	Sorafenib, artesunate, shikonin, resibufogenin, 5-FU, SM-164	Paclitaxel, VTPA, ZnO-NPs, ivermectin
	Inhibitor	Deferoxamine, deferiprone, vitamin E, ferrostatin-1, Liproxstatin-1, DHO, SRS, CA-1, cycloheximide	IAPs (XIAP, c-IAP1/2, ILP-2, NAIP, ML-IAP/livin, Z-VAD-FMK etc.), IL-2/3/4, GM/CSF	SAR405, Bafilomycin A1, Wortmannin, LY294002, Spautin1, 3-Methyladenine, hydroxychloroquine	Necrostatin-1, Necrostatin-2, NSA, Kongensin- A	Necrosulfonamide, VX765, Z-VAD-FMK, Q-VD-Oph

#### Iron Metabolism

Iron overload caused by intracellular iron metabolism disorder is an important factor that induces ferroptosis. The cellular iron metabolism involves regulation of iron absorption, storage, utilization, excretion, and some special cell iron regulatory elements.

##### Cellular Iron Absorption, Storage, and Excretion

Iron is internalized through diet absorption by duodenal enterocytes into the bloodstream (Winter et al., 2014). The circulating iron levels can be further regulated by tissue macrophages that engulf senescent red blood cells to release iron *via* ferroportin (FPN) (Winter et al., 2014). Cellular iron absorption involves the following pathways: (1) Transferrin-bound iron (TBI) pathway refers to that circulating iron binds to transferrin (Tf) and is recognized by the transferrin receptors (TfRs) to form a Fe^3+^-containing Tf/TfR1 complex that entry into cells through receptor-mediated endocytosis. TfRs are divided into two types according to their expression patterns, TfR1 and TfR2. Iron enters cells primarily *via* binding to cell surface TfR1, while TfR2 is mainly expressed in hepatocytes and erythroid precursor cells (Kawabata, 2019). The Fe^3+^-containing Tf/TfR1 complex is phagocytosed, followed by the detachment of Fe^3+^ from Tf in the acidic environment of the endosome, reduced to Fe^2+^ by six-transmembrane epithelial antigen of prostrate 3 (STEAP3) or duodenal cytochrome b (DCYTB), two metalloreductases, and then crosses the endosomal membrane enters the cytoplasm *via* divalent metal transporter 1 (DMT1) (Kawabata, 2019; Kosman, 2020). (2) non-transferrin bound iron (NTBI) pathway refers to that free iron is available as NTBI when iron levels exceed the binding capacity of available Tf. NTBI uptake at the plasma membrane involves both Zrt/Irt-related protein 14 (ZIP14) and Zrt/Irt-related protein 8 (ZIP8) on the cell membrane surface (Ji and Kosman, 2015). Subsequently, iron is transported from the endosome to the cytoplasm *via* ZIP14 and DMT1 (van Raaij et al., 2019). (3) SCARA5/TfR1-dependent endocytosis refers to that ferritin can be endocytosed by scavenger receptor class A member 5 (SCARA5) (light chain) or TfR1 (heavy chain) on the cell membrane surface and then degraded by lysosomes (Kawabata, 2019). (4) Hemoglobin-dependent pathway refers to that iron binds to haptoglobin and is endocytosed into cells by the scavenger receptor CD163, which is cleaved in the cytoplasm by heme oxygenase 1 (HO-1) to generate Fe^2+^ (Li et al., 2009). Ferritin, the major form of iron storage in organisms, is widespread in the cytoplasm, nucleus, mitochondria, and serum (MacKenzie et al., 2008). Ferritin is a spherical polymer composed of ferritin heavy chain (FTH) and ferritin light chain (FTL) that stores iron in its shell as inactive Fe^3+^, thus preventing damage due to free iron overload. FTH has ferroxidase activity, which can convert Fe^2+^ into Fe^3+^ and store it in the shell, and Fe^2+^ can flow out through the channel formed by ferritin H or L subunits (Liu et al., 2022).

The iron exportation pathway is relatively simple. Fe^2+^ is mainly transported out of cells by FPN and then oxidized to Fe^3+^ by ferroxidase. Following transferrin binding, Fe^3+^ is reabsorbed into the intracellular iron metabolism (Masaldan et al., 2019). Increased cellular iron absorption, weakened iron storage capacity, or blocked iron excretion, will finally lead to an abnormal accumulation of iron and possible ferroptosis.

##### Labile Iron Pool

Small amounts of iron exist in the free state to form the cellular labile iron pool (LIP), also considered as the cellular chelatable pool or the redox-active iron complex pool (Kakhlon and Cabantchik, 2002). Free iron in LIP is mainly found in free ferrous iron, which can bind to various ligand groups and transported by iron chaperones, such as poly(rC)-binding protein 1 (PCBP1), mediate binding to iron-requiring or iron-containing proteins and enzymes, enabling cells to meet their metabolic needs for iron (Lv and Shang, 2018; Philpott et al., 2020). LIP is homeostatic to other forms of intracellular iron regulation, while minimizing its involvement in the formation hydroxyl radicals (OH^⋅^) from hydrogen peroxide (H_2_O_2_), further reducing the occurrence of cytotoxic chemical reactions in intracellular oxygen-rich environments (Lv and Shang, 2018).

##### Mitochondrial Iron

Mitochondria are critical sites for iron utilization and accumulation. Cytoplasmic ferrous irons can be imported into the inner mitochondrial membrane (IMM) in a membrane potential-dependent manner or be transported by ferritin and traverse the IMM *via* mitoferrin 1 or mitoferrin 2 (Wang et al., 2020). Imported iron is mainly used for heme synthesis, biosynthesis of iron-sulfur clusters, and iron storage in mitochondria (Gao et al., 2021). Fe–S clusters act as cofactors in a variety of biological processes and are required for the function of enzymes related to energy metabolism, redox reactions, DNA synthesis, and other cellular physiological processes. Fe–S clusters are essential for the function of aconitine and succinate dehydrogenase in the TCA cycle and mediate functional electron transport in the respiratory complex of the electron transport chain (Paul et al., 2017). Furthermore, Fe–S clusters are important cofactors to ensure the normal functioning of key enzymes in DNA metabolism, such as DNA helicases and DNA polymerases (Puig et al., 2017). Mitochondrial ferritin (FtMt) is a specific protein that stores iron in mitochondria, and its main function is to participate in the formation of mitochondrial iron pools to maintain iron homeostasis (Campanella et al., 2009).

##### Iron Regulation

Iron regulatory proteins (IRPs), including IRP1 and IRP2, play important roles in maintaining cellular iron homeostasis. IRP regulates iron metabolizing genes transcripts by binding to the iron response element (IRE) in 3′-untranslated region (UTR) or 5′-UTR (Gao et al., 2019). When intracellular iron is low, Fe–S occupying the active site of the IRP is released, allowing DMT1 and TfR gene transcripts to bind to IRE and increase their translation, while IRPs bind to 5′-UTR-bound FPN gene transcripts inhibits its translation, thereby reducing cellular iron excretion and increasing absorption to promote intracellular free iron growth (Wei et al., 2020). The accumulated Fe^2+^ undergoes Fenton’s reaction with H_2_O_2_ to produce a large amount of OH^⋅^, one of the ROS with strong oxidative capability, which leads to the destruction of the cell membrane by oxidative damage. Regulation of iron homeostasis prevents intracellular iron accumulation by regulating iron absorption, utilization, storage, and excretion, which is one of the most important ways to prevent ferroptosis. Iron chelators, such as deferoxamine (DFO), deferiprone (DFP), and ciclopirox olamine, can suppress ferroptosis by profound depletion of intracellular iron (Eberhard et al., 2009; Nikseresht et al., 2019). Zinc protoporphyrin IX (ZnPPIX) is a specific HO-1 inhibitor that reduces Fe^2+^ produced by intracellular heme breakdown and inhibits erastin-induced ferroptosis (Gammella et al., 2015). Decreased Recombinant iron responsive element binding protein 2 (IREB2) expression can also inhibit ferroptosis by improving the intracellular storage capacity of ferritin (Kwon et al., 2015).

#### Lipid Peroxidation

Lipid metabolism is essential for ferroptosis, and lipid peroxidation induced by ROS represents the state of oxidative stress, which is a triggering factor of ferroptosis. Polyunsaturated fatty acids (PUFAs) with labile bis-allylic hydrogen atoms are especially susceptible to ROS damage (Yang et al., 2016). PUFAs and polyunsaturated acyl-tailed phospholipids (PUFA-PL) are necessary for the normal execution of ferroptosis, which can generate ROS and lipid peroxides such as malondialdehyde (MDA), 4-hydroxynonenal (4-HNE), and lipid hydroperoxides (LOOHs) through enzymatic catalysis or autooxidation (Dixon and Stockwell, 2014; Stockwell et al., 2017). In cells under ferroptotic stress, Fenton’s reaction-mediated OH^⋅^ production and lipid peroxidation generate toxic products that damage cellular proteins and nucleic acids, resulting in cellular dysfunction and even death.

The key phospholipids involved in ferroptosis are phosphatidylethanolamines (PE), including phosphatidylethanolamines-arachidonoyl (PE-AA) and phosphatidylethanolamines-adrenoyl (PE-ADA) (Kagan et al., 2017). PE-AA and PE-ADA are important substrates for lipid peroxidation. Long-chain-fatty-acid-CoA 4 (ACSL4) esterifies coenzyme A (CoA) and catalyzes AA to intermediate AA-CoA (Doll et al., 2017). In the endoplasmic reticulum, lysophosphatidylcholine acyltransferase 3 (LPCAT3) binds PUFAs to AA-COAs using PE as a substrate, forming a PE-AA-rich membrane microenvironment (Lee et al., 2021). Finally, PE-AA and PE-ADA undergo peroxidation by lipoxygenases (LOXs) to generate PUFAs, which leads to ferroptosis ultimately (Wenzel et al., 2017). LOXs, especially LOX15, are catalysts for highly selective and specific oxidation reactions of PE-AA and PE-ADA (Stoyanovsky et al., 2019). LOX15 forms a PEBP1/LOX15 complex with phosphatidylethanolamine-binding protein 1 (PEBP1), which promotes the peroxidation of PE to PE-AA-hydrogen peroxide (OOH) metabolites (Wenzel et al., 2017). High concentrations of PE-AA-OOH in cell and organelle membranes are also prone to oxidative cleavage of loosely bound iron, and electrophilic ions attack functional proteins, resulting in impaired cellular integrity and function (Anthonymuthu et al., 2021).

Lipid peroxidation inhibitors represented by the Vitamin E family can effectively inhibit the activities of LOX, ACSL4, and LPCAT3, and prevent ferroptosis by reducing the accumulation of executioners (Mabalirajan et al., 2009; Zhang et al., 2022). Furthermore, activated protein kinase (AMPK) may regulate a mitochondrial-independent mechanism of acetyl-CoA carboxylase under energy stress by reducing PUFA biosynthesis and inhibiting ferroptosis (Lee et al., 2020).

### The Regulation of Ferroptosis

**FIGURE 2:**
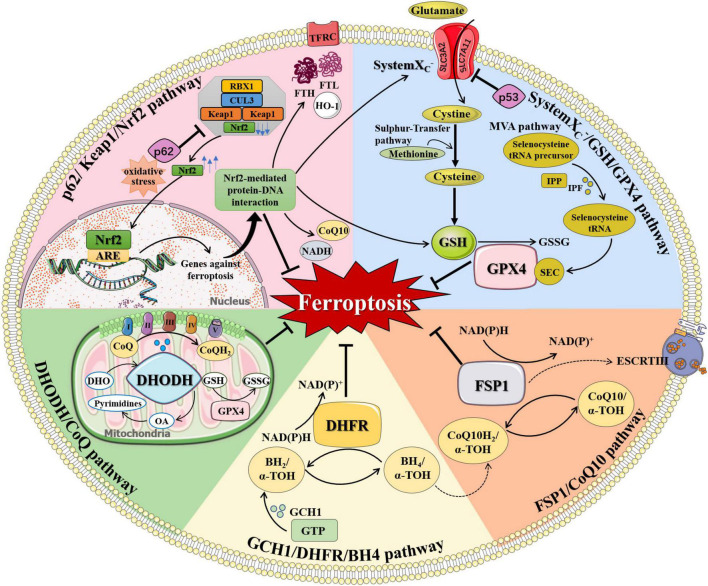
The regulation of ferroptosis. Multiple pathways inhibit ferroptosis by resisting oxidative distress and inhibiting lipid peroxidation, including systemX_*C–*_/GSH/GPX4 pathway, FSP1/CoQ10 pathway, GCH1/DHFR/BH4 pathway, DHODH/CoQ pathway, and p62/Keap1/Nrf2 pathway. (1) systemX_*C*–_/GSH/GPX4 pathway: systemX_*C*–_ consists of SLC7A11 and SLC3A2 and is capable to exchange glutamate and cystine equally. Cystine is obtained from methionine through the Sulfur-transfer pathway and then converted to cysteine intracellularly to generate GSH. Selenoprotein GPX4 detects GSH, leading to the reduction of lipid hydroperoxides to lipid alcohols and the simultaneous oxidization of two GSH into oxidized GSSG. IPP, produced by the MVA pathway, transfers isopentyl groups to isopentyl transferases-catalyzed Sec-tRNA precursors which mediate the maturation of Sec-tRNAs responsible for Sec insertion into GPX4. (2) FSP1/CoQ10 pathway: FSP1 converts CoQ10 to CoQ10H2 using NAD(P)H, which quenches lipid free radicals generated by lipid peroxidation. Membrane-associated protein complex ESCRT-III regulates membrane regeneration through membrane germination and cleavage. FSP1 transported in the plasma membrane can resist ferroptosis by enrolling ESCRT-III to activate the membrane restore mechanism. (3) GCH1/DHFR/BH4 pathway: BH4 is a potent free radical scavenger that exerts antioxidant effects in cells. The interconversion between the oxidation and reduction forms of BH4 is controlled by two enzymes, GCH1 and DHFR. The synthesis of BH4 by GCH1 expression selectively prevents PUFA-PL depletion-induced membrane lipid remodeling. DHFR is an essential enzyme for BH4 regeneration, and its inhibition may synergize with GPX4 inhibitors to induce ferroptosis. BH4 can also quench ROS by promoting the synthesis of CoQ10. (4) DHODH/CoQ pathway: DHODH is an enzyme present on the inner surface of mitochondria that catalyzes substrates DHO to produce OA. DHODH cooperates with mitochondrial GPX4 to regulate mitochondrial ferroptosis by reducing CoQ to CoQH_2_ on the mitochondrial intima independently of the cytoplasmic GPX4 or FSP1 pathways. (5) p62/Keap1/Nrf2 pathway: Under oxidative distress conditions, p62 prevents the degradation of Nrf2 in the Keap1-CUL3-RBX1 E3 ubiquitin ligase complex. Nrf2 undergoes nuclear translocation and binds to ARE to initiate transcription of multiple cytoprotective genes against ferroptosis. Nrf2-mediated protein-DNA interaction regulates the expression of FTH, FTL, FPN, TfR, HO-1, and so on for controlling cellular iron metabolism, promoting SLC7A11 expression, and increasing the production of NADPH, GSH, and CoQ10 to enhance the antioxidant capacity of cells. α-TOH, α-tocopherol; ARE, antioxidant response element; BH_2_, dihydrobiopterin; BH4, tetrahydrobiopterin; CoQ, coenzyme Q; CoQ10, coenzyme Q10; CoQH_2_, reduced coenzyme Q; CUL3, cullin 3; DHFR, dihydrofolate reductase; DHO, dihydroorotate acid; DHODH, dihydroorotate dehydrogenase; ESCRT-III, endosome sorting complex; FTH, ferritin heavy chain; FTL, ferritin light chain; FSP1, ferroptosis suppressor protein 1; GCH1, GTP cyclohydrolase-1; GPX4, glutathione peroxidase 4; GSH, glutathione; GSSG, oxidized glutathione; GTP, guanosine triphosphate; HO-1, heme oxygenase 1; IPF, isopentenyl transferase; IPP, isopentenyl pyrophosphate; Keap1, KELCH-ECH-associated protein 1; MVA pathway, mevalonate Regulation pathway; NADH, reduced nicotinamide adenine dinucleotide; NAD(P)^+^, oxidized nicotinamide adenine dinucleotide (phosphate); NAD(P)H, reduced nicotinamide adenine dinucleotide (phosphate); Nrf2, nuclear factor 2-related erythroid factor 2; OA, orotate; RBX1, RING-box protein 1; Sec, selenocysteine; SLC3A2, solute carrier family 3 member 2; SLC7A11, solute carrier family 7 member 11; TFRC, transferrin receptor.

Ferroptosis is under the regulation of multiple pathways. Besides the aforementioned iron metabolism and oxidative distress, there are anti-ferroptosis pathways that inhibit ferroptosis by inhibiting lipid peroxidation, including systemX_*C*_-/GSH/GPX4 pathway, GCH1/DHFR/BH4 pathway, FSP1/CoQ10 pathway, DHODH/CoQ pathway, and p62/Keap1/Nrf2 pathway (Figure 2). When cellular anti-ferroptosis defense pathways are interrupted, free lipid radicals are generated in the presence of free iron. It makes polyunsaturated fatty acids susceptible to lipid peroxidation, leading to irreversible oxidative distress damage to biofilms or genetic material and resulting in cell death eventually (Guichardant et al., 2011; Hadian and Stockwell, 2020).

#### SystemX_*C*_^–^/GSH/GPX4 Pathway

Among anti-ferroptosis defense pathways, the systemX_*C*_^–^/GSH/GPX4 pathway related to amino acid metabolism is widely studied. SystemX_*C*_^–^ consists of two subunits, solute carrier family 7 member 11 (SLC7A11) and solute carrier family 3 member 2 (SLC3A2). SystemX_*C*_^–^ is widely distributed in the phospholipid bilayer and play an central role in the exchange of glutamate and cystine. After entering the cell, cystine converts to cysteine, which can also be obtained from methionine through the Sulfur-transfer pathway (Zheng and Conrad, 2020). Afterward, glutathione (GSH), an important free radical scavenger in organisms, is generated from cysteine, glutamate, and glycine under the action of glutathione synthase (Lin et al., 2020; Chen L. et al., 2021). Cysteine provides sulfhydryl, an active group with antioxidant effect for GSH. Glutathione peroxidase 4 (GPX4) is a selenoprotein whose main cofactor is GSH, which reduces lipid hydroperoxides to lipid alcohols and simultaneously oxidizes two GSH to oxidized glutathione (GSSG) (Seibt et al., 2019).

The systemX_*C*_^–^/GSH/GPX4 pathway plays an important role in the downregulation of ferroptosis. Increasing extracellular glutamate concentration or decreasing cystine uptake inhibits systemX_*C*_^–^ to promote ferroptosis (Hambright et al., 2017). The classic ferroptosis inducers such as erastin and sulfadiazine block the absorption of GSH by inhibiting the systemX_*C*_^–^ (Dixon and Stockwell, 2014). p53 is a tumor suppressor protein that inhibits cystine cellular uptake by decreasing the expression of SLC7A11, resulting in reduced GPX4 activity and elevated susceptibility to ferroptosis (Jiang et al., 2015). Glutaminase 2 (GLS2), an enzyme necessary for glutamine hydrolysis is considered as an important target protein of p53. The GLS2 inhibitor compound 968 and artificial oocyte activation (AOA) inhibit erastin-induced ferroptosis (Gao et al., 2015; Jennis et al., 2016).

GPX4 is the first major regulator of ferroptosis. The compounds including RSL3, DP17, and DP110 can inhibit GPX4 directly, resulting in the accumulation of lipid peroxides and ROS for ferroptosis (Yang et al., 2014; Yang and Stockwell, 2016). The activity of GPX4 is also associated with the Mevalonate Regulation pathway (MVA pathway) (Dixon et al., 2014). Selenocysteine (Sec) is the active central amino acid of GPX4, and the Sec-tRNA is responsible for embedding Sec into GPX4. Isopentenyl pyrophosphate (IPP) produced by the MVA pathway transfers the isopentyl group to the Sec-tRNA precursor catalyzed by isopentenyl transferase, mediating the maturation of that specific tRNA (Wirth et al., 2014). The use of statins downregulates the MVA pathway, reduces IPP production, and disrupts Sec-tRNA maturation, resulting in deficiency of selenoprotein and cellular antioxidant capacity, ultimately, promoting ferroptosis (Watanabe et al., 2021).

#### GCH1/DHFR/BH4 Pathway

The GCH1/DHFR/BH4 pathway, discovered by Kraft and Soula in 2018, is a unique anti-ferroptosis mechanism independent of GPX4. Tetrahydrobiopterin (BH4) is a potent free radical scavenger that exerts antioxidant effects in cells through two enzymes, GTP cyclohydrolase-1 (GCH1) and dihydrofolate reductase (DHFR). Two enzymes are responsible for the interconversion between the oxidation and reduction forms of BH4. The synthesis of BH4 by GCH1 expression selectively prevents PUFA-PL depletion-induced membrane lipid remodeling (Kraft et al., 2020). DHFR is an essential enzyme for BH4 regeneration, and its inhibition may synergize with GPX4 inhibitors to induce ferroptosis (Soula et al., 2020). In addition, BH4 can also quench ROS by promoting the synthesis of Coenzyme Q10 (CoQ10) (Kraft et al., 2020). CoQ10/CoQ10H_2_ is an antioxidant that scavenges free radicals on cell membranes and maintains the fluidity and integrity of cell membranes.

#### FSP1/CoQ10 Pathway

The FSP1/CoQ10 pathway was discovered by Bersuker et al. (2019) and Doll et al. (2019). Ferroptosis suppressor protein 1 (FSP1) converts CoQ10 to CoQ10H_2_ using NAD(P)H, which quenches lipid free radicals generated by lipid peroxidation. FSP1 inhibits lipid peroxidation and ferroptosis independently of systemX_*C*_^–^/GSH/GPX4 pathway and still has a protective effect after GPX4 knockout (Stockwell, 2019). Homologous murine double minute 2 homolog (MDM2) and murine double minute X (MDMX) proteins are the two negative regulators of p53. Inhibition of MDM2 or MDMX increases the levels of FSP1 and CoQ10, leading to inhibition of ferroptosis in a p53-independent manner (Venkatesh et al., 2020). In addition, the endosomal sorting complex required for transport III (ESCRT-III) is a membrane-associated protein complex that regulates membrane regeneration through membrane germination and cleavage. FSP1 transported in the plasma membrane can resist ferroptosis by enrolling ESCRT-III to activate the membrane restore mechanism (Dai et al., 2020b). Activation of ferroptosis inducers such as sorafenib by regulating FSP1-ESCRT-III has been emerged as a novel anti-tumor strategy (Dai et al., 2020a).

#### DHODH/CoQ Pathway

In 2021, Mao et al. discovered a mitochondrial anti-ferroptosis defense mechanism mediated by dihydroorotate dehydrogenase (DHODH), which inhibits ferroptosis by reducing CoQ to CoQH_2_ on the IMM (Mao et al., 2021). DHODH is an enzyme present on the inner surface of mitochondria, whose substrates are dihydroorotate acid (DHO) and its reaction product is orotate (OA). DHO and OA have opposite effects on GPX4 inhibitor-induced ferroptosis. DHO exerts a protective role, while OA just the reverse. DHODH loss-of-function leads to extensive lipid peroxidation in mitochondria and ferroptosis in cancer cells with low GPX4 levels. Taken together, DHODH regulates mitochondrial ferroptosis independently of the cytoplasmic GPX4 or FSP1 pathways, while cooperates with mitochondrial GPX4.

#### p62/Keap1/Nrf2 Pathway

Nuclear factor 2-related erythroid factor 2 (Nrf2) is a major antioxidant element against oxidative distress in cells and a key transcriptional regulator of ferroptosis. Nrf2 is normally degraded by specific proteasome ubiquitination mediated by the Keap1-CUL3-RBX1 E3 ubiquitin ligase complex. The double-glycine repeat (DGR) domains of the Keap1 homodimer bind with the DLG and ETGE domains in Nrf2 (Bellezza et al., 2018). p62 direct interacts with Keap1, which inhibits the activity of Keap1 to bind Nrf2 (Komatsu et al., 2010). The release of DLG motif in Nrf2 from Keap1 blocks Nrf2 ubiquitination and degradation, therefore increasing the antioxidant capacity of cells (Bellezza et al., 2018). Activation of the p62/Nrf2/Keap1 pathway increases the antioxidant capacity of cells and inhibits the occurrence of ferroptosis (Sun et al., 2016). When stimulated by oxidative distress, Nrf2 undergoes nuclear translocation and binds to antioxidant response element (ARE) to initiate transcription of a variety of cytoprotective genes (Matsumaru and Motohashi, 2021). For example, Nrf2 regulates the expression of FTH and FTL for iron storage, FPN and TFRC for iron transport, and HO-1 for intracellular iron production, therefore controlling cellular iron metabolism (Zhao et al., 2021). Nrf2 can promote the expression of SLC7A11, glutathione synthase (GSS), and glucose-6-phosphate dehydrogenase (G6PD), which increases the production of NADPH, GSH, and CoQ10 to enhance the antioxidant capacity of cells (Shin et al., 2017; Carpi-Santos and Calaza, 2018; Dodson et al., 2019; Zheng and Conrad, 2020). Besides, Nrf2 regulates oxidative distress-induced lipid accumulation by regulating autophagy proteins including ATG5 and lipogenesis proteins such as Sterol-regulatory element binding proteins (SREBPs) (Bai et al., 2019; Sun et al., 2020).

## Emerging Links of Ferroptosis to Neurodegenerative Diseases

NDDs are a group of diseases characterized by protein intra- and extracellular deposition and gradual loss of a specific population of neurons, with progressive motor and cognitive decline (Baloni et al., 2021). There is growing evidence that ferroptosis and NDDs are inextricably linked. Currently, studies on ferroptosis in NDDs mainly focus on AD, PD, HD, and ALS. Although different NDDs have different mechanisms in disease evolution, ferroptosis is proven to be involved in all of them and characterized by altered brain iron homeostasis, dysregulation of antioxidant system and oxidative damage (Table 2).

**TABLE 2:** Pathological changes of NDDs associated with pathological features of ferroptosis.

Diseases	Ferroptosis-related Pathological Features	Potential pathological outcome	References
AD	Abnormal iron metabolism and iron deposition	Accumulation and aggregation of Aβ and Tau proteins	Ayton et al., 2020; Derry et al., 2020
		Abnormal increase in APP level and tau hyperphosphorylation and aggregation;	Altamura and Muckenthaler, 2009; McIntosh et al., 2019; Derry et al., 2020
		Microglial activation and neuroinflammation	van Duijn et al., 2017; McIntosh et al., 2019
		Neuronal death and NFT deterioration	Wan et al., 2019
	Redox imbalance and oxidative stress	Abnormal increase of Aβ oligomer and neuroinflammation	Saito et al., 2019
	Decreased GPX4 protein levels and elevated lipid peroxidation products	Hippocampal neurodegeneration	Hambright et al., 2017
PD	Oxidative distress and lipid peroxidation	Degeneration and loss of dopaminergic neurons	Dias et al., 2013; Hare and Double, 2016; Dionísio et al., 2021
	Abnormal iron metabolism and lipid peroxidation	α-synuclein aggregation	Angelova et al., 2020; Riederer et al., 2021
	Increased iron in SN	Aggregation and dopaminergic neuron death	Wang et al., 2009; Zhu et al., 2017; Zhang Y. et al., 2020
	Decreased GPX4 in SN	Dopaminergic neuron axon damage	Bellinger et al., 2011
	Decrease FPN expression	Microglia proinflammatory transformation	Zhang et al., 2014
	Iron homeostasis and iron deposition	Microglia activation and dopaminergic cell death	Wang Z. L. et al., 2022
	Down-regulation of the SLC7A11 gene and decreased SLC7A11 protein level	Neuroinflammation	Vallerga et al., 2020
	Elevated lipid peroxidation products and decreased GSH protein level	Increased Lewy bodies	Jenner et al., 1992
	DJ-1 gene mutation	Decreased GPX4 and GSH activity	Cao et al., 2020
HD	Iron overload and increased lipid peroxidation	Mitochondrial dysfunction and oxidative distress damage	Agrawal et al., 2018
	Decreased GPX4 activity	HTTP gene variants	Mason et al., 2013
	Recurrent glutamate abnormalities	Mitochondrial dysfunction	Reddy and Shirendeb, 2012
ALS	GPX4 depletion	Loss of motor neurons	Evans et al., 2022
	Changes in iron metabolizing proteins and elevated iron levels	SOD1 gene mutations	Jeong et al., 2009
	GSH/GPX4 depletion and increased lipid peroxidation	SOD1 gene mutations	Wang et al., 2021; Peng et al., 2022
MS	Decreased GSH protein level and systemXc^–^ activity	Microglial activation and neuroinflammation	Hu et al., 2019
	Glutathione deficiency and lipid peroxidation	Oligodendrocyte loss and demyelination	Jhelum et al., 2020

### Ferroptosis and Alzheimer’s Disease

Alzheimer’s disease is the most common NDD manifested as progressive decline in cognitive function, including impairment of multiple cognitive domains such as memory, executive function, and language (Tiwari et al., 2019). The typical pathologic features of AD are the presence of senile plaques (SP) formed by extracellular amyloid (Aβ) deposition and intracellular neurofibrillary tangles (NFTs) formed by hyperphosphorylated tau protein, ultimately leading to neuronal dysfunction and synapse loss (Lane et al., 2018). Multiple theories have been proposed regarding the mechanism of AD progression, which include neuroinflammation, Aβ overproduction/aggregation, tau hyperphosphorylation, neurotransmitter disorders, and mitochondrial dysfunction (Xia et al., 2021). Aging, genetic factors (e.g., altered APOE gene), environmental factors, and vascular diseases are considered as risk factors for AD (Tang et al., 2022). However, this does not appear to be fully sufficient to explain the pathogenesis of AD.

In recent years, the theory of the metal homeostasis mechanism represented by ferroptosis has been supported by increasing amounts of evidence (Hambright et al., 2017; Zhang et al., 2021). Human brain tissue from AD patients exhibits abnormal iron metabolism and glutamate levels, damaged systemX_*C*_^–^, and induced apparent lipid peroxidation (Hambright et al., 2017; Ayton et al., 2020). Iron deposition occurs in the hippocampus and the inferior temporal cortex of AD patients, and is strongly associated with loss of memory and cognitive decline (Ayton et al., 2020; You et al., 2021). Quantification of iron deposition using susceptibility-weighted imaging (SWI) indicates iron deposition in eight brain regions including prefrontal, parietal, temporal, amygdala, putamen, globus pallidus, cingulate cortex, and caudate nucleus in advanced AD patients (Ashraf et al., 2020). Being the only exit for iron excretion through cells, FPN1 has been found to be downregulated in the brains of AD patients and AD animal models to induce intracellular iron accumulation (Raha et al., 2013; Bao et al., 2021). Furthermore, they found that differentially expressed genes (DEGs) in ferroptosis were highly enriched in AD-related gene concentrations through Gene Set Enrichment Analysis (GSEA), which was consistent with the conclusion of Majerníková et al. (2021) after analysis of four datasets of AD DEGs. These literatures prompt a strong association of ferroptosis with the pathogenesis of AD.

Abnormal iron metabolism can result in post-translational production of inappropriately modified Aβ, leading to an abnormal increase in Aβ oligomers (Derry et al., 2020). Iron deposition promotes the expression and abnormal degradation of amyloid precursor protein (APP) through the intracellular IRE/IRP regulatory system, a control element for cellular iron homeostasis (Altamura and Muckenthaler, 2009; McIntosh et al., 2019; Derry et al., 2020). Abnormal elevation of divalent iron in AD brain induces tau hyperphosphorylation and aggregation, leading to the formation of NFTs (Wan et al., 2019). Amyloid plaques and NFTs therefore mediate ferroptosis-mediated neuronal death and AD-related cytotoxicity. Microglial activation and neuroinflammation are also typical changes during the pathogenesis of AD. Hippocampal microglia and astrocytes of AD patients contain high levels of ferritin (Zeineh et al., 2015). Iron accumulation induces the phenotype transition of microglia to a pro-inflammatory one and the reprogramming of cellular metabolism that reduces the Aβ clearance capacity of microglia (van Duijn et al., 2017; McIntosh et al., 2019).

Studies of ferroptosis suggest potential biomarkers for early prediction of AD. As mentioned above, abnormal iron metabolism has emerged as an important pathological characteristic. Quantitative susceptibility mapping (QSM) was used to observe the spatial co-localization of brain iron deposition and Aβ plaques in pre-AD patients, which demonstrated Aβ accumulation along with iron deposition (van Bergen et al., 2016b). Therefore, the imaging evidence of iron deposition has been proposed as an important diagnostic index of AD (Tao et al., 2014; Ayton et al., 2020). APOEε4, a risk factor for the development of AD, increases carrier susceptibility by increasing ferritin levels (Ayton et al., 2015). Cerebrospinal fluid (CSF) ferritin levels are positively associated with the risk of cognitive decline in AD patients with APOEε4 genotype, hence researchers also recommend CSF ferritin levels as a biomarker for AD (Ayton et al., 2018; Diouf et al., 2019).

Inspiringly, several studies have shown favorable therapeutic effects of ferroptosis inhibitors on AD animals (Zhang et al., 2018; Komaki et al., 2019; Rao et al., 2020; Farr and Xiong, 2021). The iron chelators DFO and DFP are widely used as specific inhibitors of ferroptosis. Preclinical evidence suggests that iron chelators modulate iron homeostasis, mitigate oxidative distress, improve cognition and behavioral outcomes in AD mice (Rao et al., 2020; Farr and Xiong, 2021). CoQ10 has been shown to inhibit ferroptosis in AD mice by inhibiting lipid peroxidation. After pretreatment of AD mice with CoQ10, MDA was decreased and oxidative distress was alleviated significantly, suggesting that CoQ10 exerts neuroprotective effects on Aβ-induced nerve damage (Komaki et al., 2019). The antioxidant alpha-lipoic acid (ALA) significantly inhibited tau-induced iron overload and reduced NFTs formation by enhancing GPX4 expression (Zhang et al., 2018). Similarly, Forsythoside A, the main component of *Forsythia suspensa* (Thunb.) Vahl, and Ginkgolide B, a terpene lactone derivative of Ginkgo biloba, also exhibited anti-AD properties *via* inhibiting ferroptosis-mediated neuroinflammation by activating Nrf2/GPX4 axis (Shao et al., 2021; Wang C. et al., 2022). Vitamin E can neutralize peroxidative free radicals, terminate lipid peroxidation, and reduce the risk of cognitive decline following high vitamin E supplementation in AD patients (Basambombo et al., 2017). CMS121, a lipoxygenase inhibitor, reduces the relative levels of fatty acids and PUFAs in AD mice and improves cognitive dysfunction by inhibiting lipid peroxidation (Everett et al., 2020). However, although ferroptosis inhibitors have exhibited promising therapeutic effects in AD animal models, there are still in lack of clinical evidence to support these findings, which is urgently needed to confirm ferroptosis as an important therapeutic target for AD.

### Ferroptosis and Parkinson’s Disease

Parkinson’s disease is the second most common NDD. Clinical symptoms of PD include motor symptoms (e.g., resting tremor, muscle rigidity, bradykinesia, and postural disorders) and non-motor symptoms (e.g., sleep disorders, depression, and cognitive dysfunction) (Parkinson, 2002). The most outstanding character of PD is the loss of dopaminergic neurons in the substantia nigra pars compacta (SNpc), resulting in striatal dopamine depletion, neuromelanin loss, and the appearance of α-synuclein (α-syn)-rich Lewy bodies in neurons (Hayes, 2019).

Numerous studies have implicated the increase of ferroptosis-associated oxidative distress and lipid peroxidative damage as important features of PD pathophysiology (Dionísio et al., 2021; Mahoney-Sánchez et al., 2021; Riederer et al., 2021). The SNpc and brainstem of PD patients have obvious iron deposition, and the iron concentration in the substantia nigra (SN) is related to the severity of the disease (Wieler et al., 2015). Animal studies also identified SN as the most susceptible brain region for iron accumulation during aging (Jia et al., 2018). The magnitude of elevated iron levels significantly correlated with the magnitude of α-syn aggregation, iron deposition, and dopaminergic (DA) neuron death in early-stage PD patients and PD animal models (Wang et al., 2009; Zhu et al., 2017; Zhang Y. et al., 2020). The studies of possible mechanisms revealed that iron combines with DA to form a potent oxide, which simultaneously produces dopamine quinine and 6-hydroxydopamine (6-OHDA). 6-OHDA can release iron from ferritin while exerting a new round of oxidative distress and neurotoxicity damage (Dias et al., 2013; Hare and Double, 2016). Meanwhile, high levels of iron in dopaminergic neurons exacerbate oxidative distress due to Fenton’s reaction (Dias et al., 2013; Hare and Double, 2016). Furthermore, total GPX4 levels are significantly reduced in the substantia nigra of PD patients (Bellinger et al., 2011). SLC7A11 was also hypermethylated in PD, severely affecting systemX_*C*_^–^ activity (Vallerga et al., 2020). Hence, the abnormal activities of systemX_*C*_^–^/GSH/GPX4 pathway in the SN have been proposed as an early event in PD pathogenesis (Jenner et al., 1992). In recent years, α-syn oligomers have been reported to induce ferroptosis by interacting with cell membranes and promoting lipid peroxidation (Angelova et al., 2020). In addition, an *in vitro* PD model further showed that FPN1 activity in SN microglia is inhibited and iron secretion is blocked, causing pro-inflammatory transformation (Zhang et al., 2014). Activation of glial cells and iron homeostasis-induced iron deposition form a “partners in crime” that alter cellular metabolic state, exacerbate oxidative distress, and induce ferroptosis on DA neurons (Wang Z. L. et al., 2022).

Due to the importance of ferroptosis in the pathogenesis of PD, iron metabolism and systemX_*C*_^–^/GSH/GPX4 pathway have been considered as promising therapeutic targets of PD. Iron chelators such as DFO have been shown to reduce oxidative distress damage and increase dopaminergic neuron activity, thereby improving motor symptoms (Do Van et al., 2016). Human brain imaging studies have shown that DFP decreases brain iron levels, alters cerebrospinal fluid ferritin, and mitigates dyskinesia (Martin-Bastida et al., 2017). Similarly, both the blockage of ferritin degradation by ferritinophagy inhibitors chloroquine and bafilomycin A1 and the application of an iron-free form of ferritin, apoferritin, have been reported to chelate excess iron to protect DA neurons against PD (Tian et al., 2020; Song et al., 2021). Moreover, NADPH oxidase inhibitor apocynin was also found to inhibit iron accumulation and lipid peroxidation, therefore ameliorating dopaminergic neurodegeneration and motor function abnormality in PD mice (Hou et al., 2019). Early-onset autosomal recessive PD is often accompanied by mutations in the DJ-1 gene. Using the antioxidant enzyme DJ-1 as a ferroptosis inhibitor can stabilize the sulfur transmission pathway, ensure the activity of GPX4 and GSH, and reduce susceptibility to ferroptosis (Cao et al., 2020). Elimination of oxidative distress is a novel therapeutic strategy. Diacetylbis (4-methyl-3-thiosemicarbazonato) copper*^II^* [Cu*^II^*(atsm)], a hypoxia-sensitive positron emission tomography imaging agent, has the greatest potential in the treatment of various NDDs (Zilka et al., 2021). *In vivo* and *in vitro* PD model experiments confirmed that Cu*^II^*(atsm)-mediated activation of Nrf2-related antioxidant enzymes improved motor and cognitive functions, protected SN cells against lipid peroxidation, and improved DA metabolism (Hung et al., 2012; Southon et al., 2020). Encouragingly, Cu*^II^*(atsm) has achieved preliminary positive treatment outcomes in a phase I clinical trial in PD patients (NCT03204929).

### Ferroptosis and Amyotrophic Lateral Sclerosis

Amyotrophic lateral sclerosis is a degenerative motor neuron disease featured by the progressive loss of motor neurons in the spinal cord, brainstem, and motor cortex, resulting in progressive muscle weakness and loss of respiratory function, leading to premature death (Vahsen et al., 2021). ALS is currently thought to be associated with gene mutation (e.g., SOD1 gene mutation), glutamate excitotoxicity, oxidative distress, immune disorders, mitochondrial dysfunction, and reduced axonal transport, but the exact etiology and pathogenesis remain unclear (Brown and Al-Chalabi, 2017; Hemerková and Vališ, 2021).

Ferroptosis has been implicated in the pathological process of ALS. Iron deposits were observed in the spinal cord, motor cortex, basal ganglia, and thalamus in ALS patients by MRI. Abnormally elevated iron levels were also found in the cerebrospinal fluid (Ignjatović et al., 2012; Kwan et al., 2012). The development of ALS is associated with increased oxidative damage caused by mutations in the free radical scavenging protein superoxide dismutase 1 (SOD1) gene. Deregulated ferritin and blocked intracellular iron efflux in neurons have been found in SOD1 transgenic mice, thereby increasing intracellular iron load (Jeong et al., 2009). SOD1 mutation also activates hypochlorous acid (HOCl)-myeloperoxidase (MPO) pathway, accelerating ROS accumulation and inhibiting GPX4 expression and thus leading to irreversible lipid peroxidation (Peng et al., 2022). Similar to SOD1 transgenic mice, other ALS animal models including TDP-43 and C9orf72 transgenic mice show GPX4 depletion and dysregulation of glutathione synthesis and iron-binding proteins as well (Wang et al., 2021). The *in vivo* studies have been corroborated by *in vitro* ALS model. hTBK1-c.978T > A mutation induces severe cell ferroptosis, which significantly inhibits the proliferation of NSC-34 cells (Zhang P. et al., 2020).

The involvement of ferroptosis in the progression of ALS has been widely examined. Gpx4 neuron inducible knockout (Gpx4NIKO) mice display ALS-like paralytic symptoms and spinal motor neuron death (Evans et al., 2022). The overexpression of GPX4 in ALS mouse models exhibits reduced systemic cytotoxicity of SOD1, late delayed disease-onset, improved motor function, and longer survival, which are associated with faster recovery from spinal motor neuron injury and repressed lipid peroxidation (Chen X. et al., 2021). Recent studies have further shown that human iPS cell-derived motor neuron (hiMN) death is associated with GPX4, which can be rescued by iron chelators and lipid peroxidase inhibitors, suggesting that iron toxicity has an important pathophysiological role in hiMN death (Matsuo et al., 2021). These evidences indicate a great contribution of ferroptosis to motor neuron degeneration in ALS.

The important role of ferroptosis in ALS also provides novel insights into the treatment and prognosis of ALS. The use of high-affinity, lipophilic iron chelator SIH results in improved spinal motor neuron survival and restored motor function (Jeong et al., 2009). Similarly, the treatment of conservative ferroptosis inhibitors such as Edaravone and DFP also exhibits promising neuroprotective effects on ALS (Devos et al., 2020). Edaravone is a clinically approved free radical scavenger for the treatment of ALS that largely inhibits ferroptosis in cystine deficiency and systemX_*C*_^–^/GPX4 inhibition, preventing devastating motor neuron damage (Spasić et al., 2020; Al-Chalabi et al., 2021). In a single-center pilot clinical study, patients treated with DFP (30 mg/kg/day) for 3 months showed improved ALS functional scores, mitigated oxidative distress, and reduced levels of cerebral iron and neurofilament light chain in the CSF. This study is the first to demonstrate the feasibility of iron chelators in the clinical management of ALS (Moreau et al., 2018). Furthermore, higher levels of baseline neurofilament light chain, 4-HNE, 8-oxo’2’-desoxyguanosine, and ferritin have been found to be independently associated with greater ALSFRS-r decline, suggesting the predictive value of these ferroptosis-related biomarkers for ALS diagnosis and prognosis (Devos et al., 2019).

### Ferroptosis and Huntington’s Disease

Huntington’s disease is an autosomal dominant NDD, which is featured by involuntary random movements, cognitive decline and personality changes (Wyant et al., 2017). In the first exon of the HTTP gene, the CAG triplet expands and repeats, the HTTP variants predispose to abnormal conformation of the HD protein, severing high levels of toxic molecules in the brain, resulting in disruption of antioxidant gene transcription, and disruption of cellular protein damage processing systems leads to neuronal degeneration and cell death due to oxidative distress (Ross and Tabrizi, 2011).

Neuronal death in HD shows the typical symptoms of ferroptosis, namely recurrent glutamate abnormalities, increased lipid peroxidation, decreased GSH, and persistent iron accumulation (Klepac et al., 2007; Agrawal et al., 2018; Mi et al., 2019). QSM revealed elevated iron levels in the caudate nucleus and putamen of the forebrain in HD patients (van Bergen et al., 2016a). Typical phenotypes of ferroptosis with reduced GSH levels and GPX4 activity, massive iron accumulation in neurons and lipid peroxidation were found in HD animal models (Reddy and Shirendeb, 2012; Mason et al., 2013). *In vitro* models also demonstrated the importance of ferroptosis in HD pathogenesis as ferrostatin-1 effectively prevented ferroptosis in HTT exon-overexpressing cells (Skouta et al., 2014).

Inhibition of ferroptosis is beneficial in impeding the pathological progression of HD. Mounting studies have demonstrated that CoQ10 supplementation for HD patients can improve mitochondrial function and alleviate membrane lipid peroxidation caused by oxidative distress (Andrich et al., 2004). CoQ10 supplementation in R6/2HD mice had neuroprotective effects on motor performance and prolonged survival of HD mice, by alleviating the reduced glutathione production, lipid peroxidation and oxidative DNA damage (Yang et al., 2009). Besides, targeting iron and activating Nrf2-mediated pathway can be potential therapeutic strategies for HD (Mi et al., 2019). Both the activation of Nrf2 by the cyanoenone triterpenoids CDDO-ethyl amide/CDDO-trifluoroethyl amide and the disruption of Keap1-Nrf2 interaction by KEAP1-modifying small molecule MIND4-17 significantly enhance antioxidant functions of brain cells and ameliorate the behavioral phenotype in HD mouse (Stack et al., 2010; Quinti et al., 2017). Therefore, the above evidences demonstrated a key role of ferroptosis in the pathogenesis of HD, suggesting ferroptosis as an important target for treating and/or preventing HD.

### Ferroptosis and Multiple Sclerosis

Multiple sclerosis is a chronic autoimmune disease of the human central nervous system (CNS), characterized by neuroinflammation, demyelination, oligodendrocyte loss, and neurodegeneration (Lucchinetti et al., 1996). Recent progress in understanding the pathogenesis of MS suggests major roles for microglia in the disease, in which microglia alter their transcriptional profile and activate into profuse inflammatory phenotypes (Voet et al., 2019).

Pioneer studies were carried out to unveil the association of ferroptosis with microglia-driven neuroinflammation and MS. Hu et al. (2019) reported reduced GPX4 expression levels in the gray matter of MS patients and in the spinal cord of experimental autoimmune encephalomyelitis (EAE) mice, a classic MS mouse model. Further studies demonstrated that ferroptosis mediated rapid loss of oligodendrocytes and demyelination induced by cuprizone, a widely used copper chelator to induce MS-like pathological phenotype (Jhelum et al., 2020). The inflammatory responses of microglia *in vitro* can be triggered by ferroptosis and lipopolysaccharides (LPS)-stimulated systemic inflammation *in vivo* can be partially blocked by inhibiting ferroptosis *via* Ferrostatin-1 treatment (Cui et al., 2021). Interestingly, multiple publications suggested ferroptosis resistance of microglia. Pro-inflammatory microglia display higher ferroptosis resistance than alternatively activated anti-inflammatory ones highly likely due to their abundant expression of NRF2 and enrichment of iNOS/NO^⋅^ (Jhelum et al., 2020; Kapralov et al., 2020). Pro-inflammatory microglia, but not anti-inflammatory ones, promote distant suppression of ferroptosis, implying anti-ferroptotic effects of neuroinflammation. Therefore, the exact roles of ferroptosis in MS remain unclear.

Besides, literatures have implied the involvement of ferroptosis in the pathogenesis of other NDDs. For example, ferroptosis has been found in light-induced retinal degeneration and Ferrostatin-1 has been reported to protect retina against degeneration *via* elevating the expression of SLC7A11 and GPX4 protein expression and suppress neuroinflammation (Tang et al., 2021). However, the pathological roles of ferroptosis in these unmentioned NDDs remain vague, which requires further investigations to clarify.

Hence, ferroptosis has been emerged as a key contributor to the pathogenesis of various NDDs. Given that, ferroptotic factors and ferroptosis-related signaling pathways have now been considered as potential diagnostic/prognostic biomarkers and therapeutic targets of NDDs. It is also worth-noting that ferroptosis has been reported to participate in neuroinflammation and neuronal death post-acute brain damage, which may play a role in the pathogenesis of NDDs as well (Mao et al., 2020; Cao et al., 2021; Cui et al., 2021; Ge et al., 2022; Qu et al., 2022).

## Conclusion

In summary, NDDs are a group of pathologically and clinically heterogeneous diseases that create a heavy burden on healthcare systems, families, and individuals. However, the pathogenesis of NDDs has not been fully elucidated. To date, sufficient evidence has demonstrated that ferroptosis is closely related to the occurrence and progression of NDDs. The underlying mechanisms involve altered activities of Gpx4- and Nrf2-mediated signaling pathways, the deregulation of iron metabolism, oxidative distress, and glutamate-mediated excitotoxicity. In the future, more comprehensive investigations will greatly expand our understanding on the roles of ferroptosis in the pathogenesis of NDDs and shed light on the development of novel therapeutic technologies and strategies for treating incurable NDDs.

## Author Contributions

JL, JH, and JZ conceived the manuscript. YS and JL collected the references. YS, XX, DB, JL, and JH wrote the manuscript. All authors read and approved the final manuscript.

## Conflict of Interest

The authors declare that the research was conducted in the absence of any commercial or financial relationships that could be construed as a potential conflict of interest.

## Publisher’s Note

All claims expressed in this article are solely those of the authors and do not necessarily represent those of their affiliated organizations, or those of the publisher, the editors and the reviewers. Any product that may be evaluated in this article, or claim that may be made by its manufacturer, is not guaranteed or endorsed by the publisher.

## Glossary

4-HNE: 4-hydroxynonenal
5-FU: 5-fluorouracil
6-OHDA: 6-hydroxydopamine
AA: arachidonoyl
ACSL4: long-chain-fatty-acid-CoA 4
AD: Alzheimer’s disease
ADA: adrenoyl
ALA: alpha-lipoic acid
ALS: amyotrophic lateral sclerosis
AKT: threonine protein kinase
AMPK: activated protein kinase
APP: amyloid precursor protein
ARE: antioxidant response element
ATG: autophagy-related
ATP: adenosine triphosphate
Aβ: amyloid β
Bax: Bcl2-associated X.

Bcl-2: B-cell lymphoma-2
BH_2_: dihydrobiopterin
BH_4_: tetrahydrobiopterin
CDDO: 2-cyano-3,12-dioxooleana-1,9(11)-dien-28-oic acid
c-IAP 1/2: cellular inhibitor of apoptosis proteins ½
CIL56: caspase-independent lethal 56
CNS: central nervous system
CoA: coenzyme A
CoQ10: coenzyme Q10
CoQH_2_: reduced coenzyme Q
CSF: cerebrospinal fluid
Cu*^II^*(atsm): diacetylbis (4-methyl-3-thiosemicarbazonato) copper*^II^*
CUL3: cullin 3
DA: dopaminergic
DAMPs: damage-associated molecular patterns
DCYTB: duodenal cytochrome b
DFO: deferoxamine
DEGs: differentially expressed genes
DFP: deferiprone
DGR: double-glycine repeat
DHFR: dihydrofolate reductase
DHO: dihydroorotate acid
DHODH: dihydroorotate dehydrogenase
DMT1: divalent metal transporters 1
DNA: deoxyribonucleic acid
Ecto-CRT: surface-exposed calreticulin
EAE: experimental autoimmune encephalomyelitis
ERK: extracellular signal-regulated kinase
ESCRT-III: endosomal sorting complex required for transport III
FASL: factor associated suicide ligend
Fe^2+^: ferrous iron
Fe^3+^: ferric iron
Fe–S: iron–sulfur clusters
FIN56: ferroptosis inducing 56
FINO_2_: 1,2-dioxolane
FPN: ferroportin
FSP1: ferroptosis suppressor protein 1
FTH: ferritin heavy chain
FTL: ferritin light chain
FtMt: mitochondrial ferritin
G6PD: glucose-6-phosphate dehydrogenase
GCH1: GTP cyclohydrolase-1
GLS2: glutaminase 2
GM/CSF: granulocyte-macrophage colony-stimulating factor
GPX4: glutathione peroxidase 4
Gpx4NIKO: Gpx4 neuron inducible knockout
GSDMD: gasdermin D
GSEA: gene set enrichment analysis
GSH: glutathione
GSS: glutathione synthase
GSSG: oxidized glutathione
H_2_O_2_: hydrogen peroxide
HD: Huntington’s disease
HMGB1: high mobility group box 1 protein
HO-1: heme oxygenase 1
HOCl: hypochlorous acid
IAPs: the inhibitor of apoptosis proteins
IL-2/3/4: interleukin-2/3/4
ILP-2: inhibitor of apoptosis protein-like protein-2
IMM: inner mitochondrial membrane
IPF: isopentenyl transferase
IPP: isopentenyl pyrophosphate
IRE: iron-responsive element
IREB2: iron responsive element binding protein 2
IRP: iron-regulatory protein
Keap1: KELCH-ECH-associated protein 1
LIP: labile iron pool
LOOHs: lipid hydroperoxides
LOX: lipoxygenase
LPCAT3: lysophosphatidylcholine acyltransferase 3
LPS: lipopolysaccharides
MAPK: mitogen-activated protein kinase
MDA: malondialdehyde
MDM2: murine double minute 2 homolog
MDMX: murine double minute X
MLKL: mixed lineage kinase domain-like protein
MPO: myeloperoxidase
MRI: magnetic resonance imaging
MS: multiple sclerosis
mTOR: mammalian target of rapamycin
MVA pathway: Mevalonate Regulation Pathway
NAD(P)^+^: oxidized nicotinamide adenine dinucleotide (phosphate)
NAD(P)H: reduced nicotinamide adenine dinucleotide (phosphate)
NADH: reduced nicotinamide adenine dinucleotide
NDDs: neurodegenerative diseases
NFTs: neurofibrillary tangles
NLRP3: NLR family pyrin domain-containing protein 3
Nrf2: nuclear factor 2-related erythroid factor 2
NSA: necrosulfonamide
NTBI: non-transferrin bound iron
OA: orotate
OH^⋅^: hydroxyl radical
PARP-1: Poly (ADP-ribose) polymerase 1
PCBP1: poly(rC)-binding protein 1
PD: Parkinson’s disease
PE: phosphatidylethanolamines
PE-AA: phosphatidylethanolamines-arachidonoyl
PE-AA-OOH: hydroperoxides of phosphatidylethanolamines-arachidonoyl
PE-ADA: phosphatidylethanolamines-adrenoyl
PE-ADA: phosphatidylethanolamines-adrenoyl
PE-ADA-OOH: hydroperoxides of phosphatidylethanolamines- adrenoyl
PEBP1: phosphatidylethanolamine-binding protein 1
PI3K: phosphoinositide 3-kinase
PUFAs: polyunsaturated fatty acids
PUFA-PL: polyunsaturated acyl-tailed phospholipids
RCD: regulated cell death
RIP-1: receptor interacting protein-1
RIP-3: receptor interacting protein-1
ROS: reactive oxygen species
RSL3: RAS-selective lethal 3
SAS: sulfasalazine
SCARA5: scavenger receptor class A member 5
Sec: selenocysteine
SLC3A2: solute carrier family 3 member 2
SLC7A11: solute carrier family 7 member 11
SM-164: Smac mimetics-164
SOD1: superoxide dismutase
SN: substantia nigra
SP: senile plaques
SREBPs: sterol-regulatory element-binding proteins
STEAP3: six-transmembrane epithelial antigen of prostrate 3
SWI: susceptibility-weighted imaging
TBI: transferrin-bound iron
TCA cycle: tricarboxylic acid cycle
Tf: transferrin
TfR: transferrin receptor 1
TGF-β: transforming growth factor beta
TRAIL: tumor necrosis factor- related apoptosis-inducing ligand
tRNA: transfer ribonucleic acid
UTR: untranslated region
VTPA: virus-spike tumor-activatable pyroptotic agent
XIAP: X-linked inhibitor of apoptosis
ZIP14: Zrt/Irt-related protein 14
ZIP8: Zrt/Irt-related protein 8
ZnO-NPs: zinc oxide nanoparticles
ZnPPIX: zinc protoporphyrin IX
α-Syn: α-synuclein
α-TOH: α-tocopherol
Δψm: mitochondrion membrane potential.
